# Peach Palm (*Bactris gasipaes*) as a Sustainable Source of Plant Proteins, Dietary Fiber and Other Functional Ingredients: Recovery Techniques and Functional Food Applications

**DOI:** 10.3390/foods15040736

**Published:** 2026-02-16

**Authors:** Kartik Sharma, Nattaya Konsue, Samart Sai-Ut, Ekasit Onsaard, Wanli Zhang, Shusong Wu, Jia-Qiang Huang, Young Hoon Jung, Saroat Rawdkuen

**Affiliations:** 1 Unit of Innovative Food Packaging and Biomaterials, School of Agro-Industry, Mae Fah Luang University, Chiang Rai 57100, Thailand; kartik.coa@gmail.com (K.S.); nattaya.kon@mfu.ac.th (N.K.); 2 Department of Food Science, Faculty of Science, Burapha University, Chonburi 20131, Thailand; samarts@go.buu.ac.th; 3 Department of Agro-Industry, Faculty of Agriculture, Ubon Ratchathani University, Ubon Ratchathani 34190, Thailand; ekasit.o@ubu.ac.th; 4 School of Food Science and Engineering, Hainan University, Haikou 570228, China; zwl@hainanu.edu.cn; 5 College of Animal Science and Technology, Hunan Agricultural University, Changsha 410128, China; wush688@hunau.edu.cn; 6 Department of Nutrition and Health, China Agricultural University, Beijing 100083, China; jqhuang@cau.edu.cn; 7 School of Food Science and Biotechnology, Kyungpook National University, Daegu 41566, Republic of Korea; younghoonjung@knu.ac.kr

**Keywords:** peach palm, plant-based foods, protein isolate, bioactive compounds, waste valorization

## Abstract

The current rise in global population and the subsequent demand for food supply to meet the current population has directed the attention of researchers towards sustainable, plant-based sources, particularly underutilized crops. *Bactris gasipaes* is one such underutilized crop with significant functional food value. During processing, 84% of the total weight of the palm is discarded in the form of waste, or so-called by-products, which are a rich source of bioactive compounds. These compounds can be effectively recovered through modern extraction and valorization techniques. This review critically examines the extraction methods, nutritional profiles, and valorization opportunities of peach palm, highlighting both traditional uses and innovative processing strategies. Recent advances enable the targeted recovery of multiple peach palm fractions, e.g., proteins are commonly extracted using alkaline methods, lipids and carotenoids via green solvents or supercritical CO_2_, and starch and dietary fiber through hydrothermal or downstream separation processes. Key nutritional findings demonstrate that peach palm fractions offer significant protein content (with isolates reaching 40 to 60%), a favorable starch profile (up to 79%), and abundant unsaturated lipids and carotenoids, making them suitable for gluten-free, protein-enriched, and functional ingredient applications. Previous studies have focused mainly on the edible pulp of peach palm for protein, lipid, and carotenoid extraction, whereas other fractions such as peel, seed, and processing residues remain comparatively underexplored due to technological and safety constraints. This review provides a consolidated and critical overview of recent advances in fractionation and green extraction strategies for multiple value-added streams (proteins, lipids, carotenoids, starch, and dietary fiber), highlighting knowledge gaps and opportunities for sustainable food ingredient development.

## 1. Introduction

**Figure 1:**
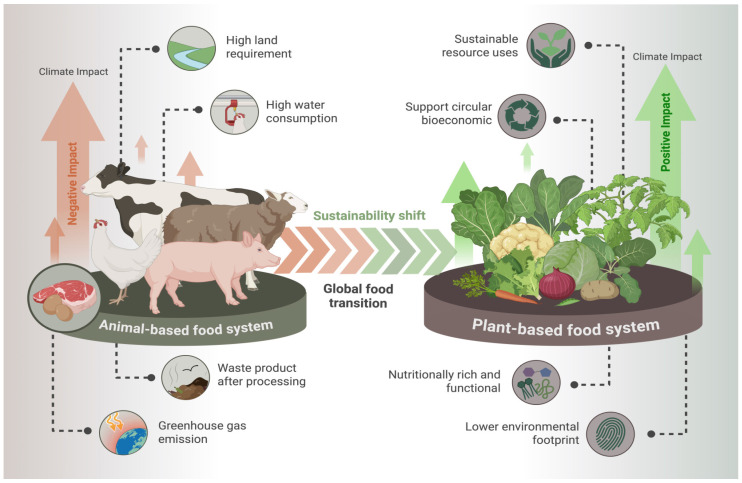
Sustainability-driven transition in global food systems from animal-based to plant-based production. https://biorender.com/2btt43z. Accessed on 5 February 2026.

The alarming rise of global population, which is projected to reach up to 10 billion within the next 25 years, has emerged as an urgent challenge to ensure adequate and sustainable nutrition for all. This escalating figure demands an estimated 70% increase in food production to meet future needs [1]. The growing demand for food is not only driven by population expansion but also by increasing consumer awareness towards sustainable and plant-based foods. While animal-derived foods are nutritionally rich, their production is associated with considerable environmental burdens. This includes high land and water usage with elevated greenhouse gas emissions [2]. As a result, global priorities are shifting more towards sustainable, plant-based foods rather than animal-based sources. This trend has directed researchers’ interest towards underutilized tropical crops that possess higher nutritional and functional properties [3]. Figure 1 depicts the sustainability shift from animal-based to plant-based food systems, emphasizing environmental impacts and the circular bioeconomy benefits associated with plant-based diets.

*Bactris gasipaes*, commonly known as *Pupunha*, belonging to the *Arecaceae* family, is one such underutilized crop with significant potential for valorization in future food systems [4]. Its leaves, fruit, seeds, roots, and wood have been used traditionally for various purposes. For instance, the fruit is used to cure body aches, headaches, anti-inflammatory for gallbladder and eyes; leaves are employed for curing epilepsy; seeds are used in stomach aches; roots are used during urinary or menstrual problems, uterine infections, and many more [5]. Nutritionally, the fruit is a rich source of dietary fiber, carbohydrates, fats and bioactive compounds. The fruit possesses less protein but contains all essential amino acids [5]. It is also rich in essential minerals, with potassium, selenium and chromium being the dominating ones.

The fruits and by-products, which are dumped directly into the environment after processing, are rich in nutrients and bioactive compounds. These compounds can be effectively recovered through modern extraction and valorization techniques. Such strategies mirror the broader shift of the food industry toward circular bioeconomy principles, emphasizing clean label formulation and the expansion of plant-based protein alternatives. Notably, the valorization of residual fractions, including basal segments and both inner and outer sheaths, offers pathways for zero-waste processing, functional ingredient development, and integration within biorefinery systems [6].

Recent progress in technologies and advancements in extraction technologies has opened numerous ways to fully harness the potential of peach palm, including its by-products. Following protein fractionation, functional optimization can enable its incorporation into meat analog formulations, while starch fractions can be extracted and modified for texturizing or prebiotic purposes. Likewise, lipid and carotenoid isolates serve as natural colorants and antioxidant-rich ingredients in clean label food systems. However, several technical challenges remain, including the need to improve extraction efficiency, address safety risks linked to calcium oxalate crystals, and enhance the techno-functional performance of recovered compounds to satisfy industrial processing standards.

While our previous review ‘Unlocking the potential of peach palm for plant-based foods’ comprehensively summarized the nutritional composition, processing effects, and general food applications of peach palm, the present review is intentionally focused on extraction, fractionation, and valorization strategies for generating ingredient-grade fractions. Emphasis is placed on processing pathways, green extraction technologies, and the functional potential of isolated proteins, lipids/carotenoids, starch, and dietary fiber for sustainable food systems.

Therefore, the aim of the current review is to critically examine the current knowledge on the extraction, processing and valorization of peach palm and to explore its potential in future food applications. The discussion focuses on strategies for recovering high-value compounds from both fruits and processing by-products, evaluates their applicability in plant-based food systems, and identifies persistent research gaps and technological challenges. By integrating technological, nutritional, and sustainability perspectives, the review positions peach palm as a promising circular raw material capable of contributing to the evolving sustainable food industry.

## 2. Processing Potential for Plant-Based Food Applications

**Figure 2:**
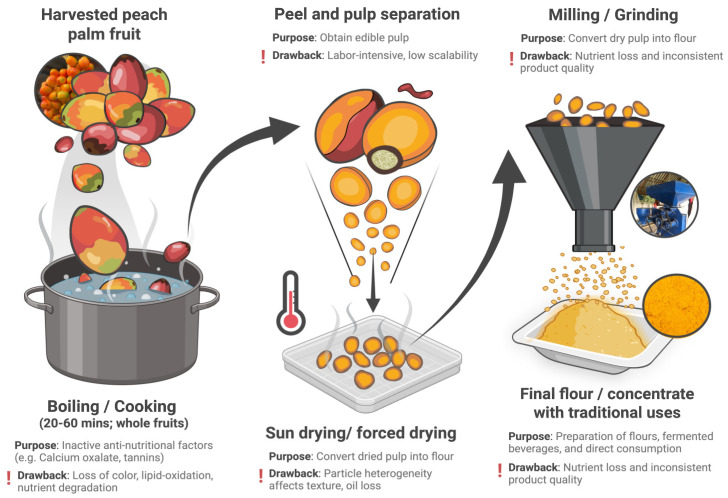
Traditional processing of peach palm fruit: main steps, purposes, and drawbacks. https://biorender.com/3axwi6v. Accessed on 5 February 2026.

Peach palm has been used traditionally for various applications, making it a promising species for diversifying and modernizing plant-based food systems. The application of modern technologies can facilitate its scalability and sustainable processing. Historically, peach palm was consumed after cooking in the form of cooked fruit, slowly fermented silage and fermented chichi. This process inactivates the antinutritional factors, including phytates, trypsin inhibitors, tannins and majorly calcium oxalate crystals [5]. As the effects of traditional processing on nutritional quality and antinutritional factors have been comprehensively reviewed in our previous review, the current discussion is limited to aspects relevant to downstream ingredient processing and functionality. Cooking peach palm softens the texture of the fruit, thereby making the pulp suitable for direct consumption or for preparing flour. The flour thus obtained has attracted attention as a gluten-free ingredient for use in bakery applications, broadening the dietary choices for individuals with celiac disease or gluten intolerance. Functionally, peach palm flour shows high water- and oil-holding capacity, strong emulsifying ability, and good stability in complex food matrices. Nevertheless, conventional processing treatments can influence lipid and fiber content, promote Maillard reaction browning, and alter the sensory characteristics of the product. Such changes ultimately affect the nutritional quality and techno-functional performance of the resulting flours or starches [7]. Figure 2 illustrates the conventional processing pathway of peach palm fruit, highlighting each stage, from harvesting and boiling to drying and milling, along with its main purpose and associated drawbacks.

Different types of peach palm vary in terms of varieties, ecotypes, and physical and biochemical composition. Therefore, there is a need for industrial-scale utilization and product development, which will offer consistency in the product. According to González-Jaramillo et al. [5], adapting processing methods to the intrinsic properties of peach palm varieties, including starch and carotenoid content, is key to consistent and innovative product development. In addition, it was also observed that the optimization of hydrothermal and drying parameters enhances product safety and functionality during industrial production or large-scale manufacturing operations [5,7].

The processing strategies summarized in Table 1 illustrate the transition from conventional thermal treatments toward greener and more selective technologies for peach palm valorization. Although traditional processing ensures detoxification and basic functionality, the integration of green technologies is required to enhance both functional and economic value. Microwave- and ultrasound-assisted extractions have been reported to improve carotenoid and phenolic recovery from pulp and peel while reducing solvent consumption, thereby offering advantages in efficiency and sustainability [8,9]. Ionic liquid systems further demonstrate high selectivity and bioactive yields suitable for functional ingredient development, although their broader application requires careful solvent management [10]. In parallel, supercritical CO_2_ extraction combined with targeted physicochemical starch modification enables the design of ingredients with tailored techno-functional properties, such as low-digestibility starches for glycemic control and carotenoid-rich oils for natural coloration and antioxidant applications [11,12]. Collectively, these technological developments support the production of functional foods that align with the increasing consumer demand for health-oriented and sustainable products [5].

### 2.1. Protein Extraction and Fractionation Potential

(a) Theoretical protein concentrate/isolate development

Peach palm has the potential of being a promising source of plant-based proteins. Peach palm’s several anatomical parts, most notably the fruit pulp, derived flour, and palm heart, contain a substantial number of proteins with balanced essential amino acid profiles. The protein contents in peach palm pulp ranges from 1.8 to 4.6 g/100 g on a dry basis, with higher values reported after dehydration and milling into flour, as reported in various studies. Evidence from Soares et al. [13] indicates that the protein content in peach palm flour can reach up to 20% on a dry matter basis. The high protein content in peach palm in comparison with other tropical fruits and starchy staples provides a strong basis for the production of protein concentrates and enrichment using peach palm. The development of protein concentrates and isolates typically entails mechanical disruption (grinding or milling), followed by aqueous or alkaline extraction and isoelectric precipitation or ultrafiltration to recover discrete protein fractions [16,17]. Some of the existing literature highlights that the nutritional value of flour derived from the fruit pulp and peel of peach palm is not only relatively rich in protein but also contains all essential amino acids in amounts that meet or exceed FAO reference patterns [18,19]. This unique nutritional profile of peach palm makes it suitable not just as a nutritional fortifier but also as a base for functional isolates with balanced amino acid profiles.

(b) Functional protein modification opportunities

Functional protein modification aims to improve solubility, water-/oil-binding capacity, emulsification and foaming properties to expand their value in plant-based food systems [4,7,13]. Recent studies emphasize several innovative methods, including enzymatic hydrolysis, physical processes and thermal treatments to improve the techno-functional profile of peach palm proteins [13,15]. Enzymatic hydrolysis is one of the approaches to produce bioactive peptides with various functional and biological properties [20,21]. This has been well documented in various legumes and oil seeds, where enzymatic hydrolysis significantly enhanced the digestibility and solubility of the resulted peptides along with augmented antioxidant and antihypertensive activities [22,23]. Enzymatic hydrolysis is responsible for altering the size of the peptides and this in turn improves their emulsification properties. Consequently, such processes provide more ways for their incorporation into vegan dairy products, baking formulations, and beverage systems [5,13]. Physical processes like ultrasonication and high-pressure processing (HPP) and thermal treatments such as hydrothermal treatment also provide opportunities for improving functional properties by altering the shape of proteins [24]. Justino et al. [25] demonstrated that the use of ultrasound technology results in an effective breakdown of protein aggregates and a reduction in particle size. This results in enhanced dissolution and greater surface coverage. These properties make proteins suitable as natural stabilizers for emulsified and aerated systems [25]. Meanwhile, HPP causes compact protein structures (globular shape) to unfold, thereby exposing the hydrophobic domains that increase both foaming and emulsifying capacities [26]. Overall, enzymatic treatments mainly improve digestibility and bioactivity, physical processes (ultrasound and high-pressure processing) enhance solubility and interfacial properties, controlled chemical reactions influence flavor and thermal stability, and fermentation-based approaches reduce antinutritional factors while generating novel functional attributes.

Chemical modification offers another approach for altering protein functionality when conducted under controlled food-processing conditions. Non-enzymatic glycation (Maillard-type reactions) may occur between amino groups of proteins and carbonyl groups of reducing sugars during thermal treatment, influencing both functional properties and flavor development [21,27]. However, such reactions are not considered deliberate protein modification methodologies, as uncontrolled Maillard reactions can lead to nutritional losses and the formation of advanced glycation end products. Previous studies have shown that mild and controlled glycation can improve solubility and thermal stability in proteins from tropical and Amazonian crops [28,29]. In this context, carefully regulated glycation reactions could potentially be explored for peach palm proteins to modulate functional performance in plant-based formulations, while avoiding excessive thermal damage.

**Figure 3:**
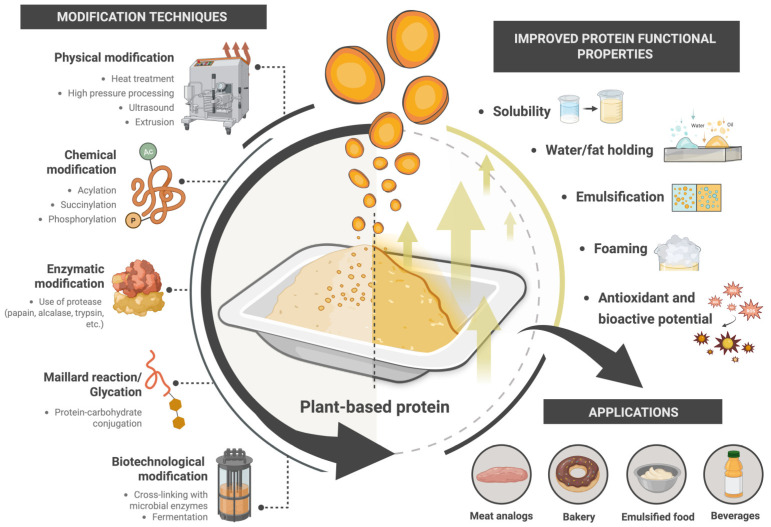
Functional modification strategies to improve techno-functional properties of plant proteins. https://biorender.com/p7zdxpo. Accessed on 5 February 2026.

Overall, these diverse protein enhancement techniques, including enzymatic treatment, physical processing, chemical modification and fermentation-based methods, allow for the precise improvement of peach palm proteins. Each approach offers a way to adjust their structures or functions to suit modern food production needs. As a result, these proteins are finding a wider use in sustainable, plant-based foods that demand both performance and quality. Table 2 summarizes the recent advances in protein extraction and functional modification techniques from peach palm, whereas Figure 3 summarizes major physical, chemical, enzymatic and biotechnological modification strategies used to enhance the functional properties of plant-derived proteins, including solubility, emulsification and bioactivity.

### 2.2. Starch and Carbohydrate Utilization

Peach palm possesses high starch concentrations. Rosário et al. [35] reported that peach palm contains about 79% total starch, with approximately 12.4% amylose and 66.6% amylopectin in the fruit matrix. The low amylose content in peach palm aids in the production of starch with desirable properties. Therefore, such flours can be used for various food and industrial purposes where variable mechanical or functional properties of starch is required for product formulations [35]. The native starches from peach palm show varied granule shapes and sizes; however, this variation is strongly influenced by the maturation stage of the fruit. As such, starches from unripe fruits do not show distinct morphologies [4,35,36].

(a) Starch isolation and modification

Peach palm starch is obtained from the peach palm pulp. It contains residual levels of ash, proteins, lipids and fibers. Starch extraction involves isolating starch granules from plant tissues through processes such as grinding, steeping, sieving, and centrifugation. The separated starch is then washed and dried to achieve high purity and yield [36]. Extraction methods like wet milling and alkaline treatment can influence starch quality, purity, and functional properties by affecting its molecular structure and amylose-to-amylopectin ratio. Extraction techniques, whether mechanical, chemical, or physical, are selected based on the raw material and the desired characteristics of the final product. Each method presents a trade-off between yield, purity, and functional properties [37,38].

The processing methods entail the extraction and alteration of starch, as the native form of starch is not suitable for direct use in industrial applications due to its unfavorable characteristics such as propensity towards retrogradation, high viscosity even at low concentrations, handling problems, poor freeze–thaw stability, low process tolerance, and gel opacity, which prevent its use in food processing [39]. To enhance starch’s physicochemical and functional properties to meet industrial standards, it must undergo various modifications, such as physical, chemical, and enzymatic modifications. Chemical modification is highly effective, but it does have disadvantages, such as the high cost of chemical residues, which makes it environmentally unfriendly. On the other hand, enzymatic methods are more intricate and time-consuming [40]. Conversely, physical modification techniques lack chemicals and do not produce wastewater with toxic residues. Furthermore, the wastewater treatment required after modification is minimal. Modern physical modification techniques such as cold plasma processing, irradiation, and hydrothermal treatment are used in the starch industry to produce starch with direct commercial applications that negatively impact the environment and consumer consumption. The experimental studies demonstrate that treatments like annealing, hydrothermal treatment (HMT), autoclaving, and cross-linking with citric acid can significantly improve starch characteristics such as crystallinity, thermal stability, and resistance to retrogradation. Thus, with the aid of these techniques, the starch of peach palm can be used in starch-based food systems and for developing biodegradable materials. For example, Soares et al. [4] reported the production and characterization of a peach palm starch-based biodegradable thermoplastic. This thermoplastic showed high tensile strength along with high thermal degradation ability. Consequently, it can be concluded that peach palm starch may serve as an important environmentally friendly alternative in the production of this type of material, which is of great interest to the packaging industry [40,41,42].

(b) Fiber extraction and functionality

Peach palm pulp is classified as a fiber-rich food [4]. Dietary fiber plays an important role in food systems by enhancing food texture through water retention and supporting digestive health, functional properties, and nutritional quality. The key dietary fiber components, viz. cellulose, hemicellulose, and lignin, have been successfully obtained from the processing discards or by-products. Dietary fibers are not digested and absorbed in the human small intestine. They are threatened by complete or partial fermentation in the large intestine. Dietary fibers have beneficial physiological functions, including laxation and improving bowel health by stimulating the growth of beneficial gut micro-flora, lowering blood cholesterol and glucose levels, preventing obesity, coronary heart diseases, diabetes, blood pressure, and lowering energy intake [15]. Insoluble dietary fibers remain intact during digestion and are involved in reducing the risk of coronary heart disease and type 2 diabetes. Giombelli et al. [42] recovered dietary fiber concentrates from peach palm waste using subcritical water and low-pressure aqueous systems as extraction techniques. The fibers obtained using these methods possess high water- and oil-holding capacities. Under subcritical extraction conditions, the fibers become more fragmented and exhibit higher porosity. The substantial cellulose composition, combined with altered conformational attributes detected via FT-IR analysis, leads to improved hydration and emulsification capabilities, thereby expanding the potential applications of peach palm fibers in functional food systems. Furthermore, Giombelli et al. [42] reported that scanning electron microscopy revealed a more open, porous microstructure in starch after subcritical water treatment, which enhanced its functional properties, including oil-binding capacity and swelling index.

(c) Prebiotic and functional carbohydrate applications

The carbohydrates in peach palm, mainly its soluble dietary fiber and processed starches, exhibit prebiotic potential. Šárka et al. [43] observed that pectic polysaccharides extracted from peach palm stimulate beneficial gut microbial fermentation in vitro. Their resistance to enzymatic degradation further enhances their prebiotic activity. Likewise, [44] also demonstrated that resistant starch fractions produced through controlled modification digest slowly and promote favorable fecal fermentation profiles. In addition to these components, xylooligosaccharides (XOS), composed of 2–20 D-xylose units linked by β (1 → 4) glycosidic bonds, can also contribute to prebiotic functionality. XOS are typically derived from the hydrolysis of xylan present in lignocellulosic biomass, and, because humans lack the enzymes to hydrolyze β-linkages, these oligosaccharides reach the large intestine intact, where they serve as substrates for beneficial microbes such as Bifidobacterium and Lactobacillus [15]. Their fermentation not only supports gut microbial balance but also provides secondary health benefits, including improved calcium absorption, better lipid metabolism, and reduced risk of chronic metabolic disorders. Collectively, these dietary components play vital roles in blood sugar regulation, colon health, and in sustaining probiotic bacteria that help maintain a balanced intestinal microbiome [40].

### 2.3. Lipid and Bioactive Compound Extraction

(a) Oil extraction for food applications

Considering that the peach palm fruit generally has a high lipid content, it is important to consider its lipid profile, particularly the quantity and quality of the fatty acids present in the pulp. The fruit of peach palm is naturally rich in oils, making it a promising source for the production of edible oil. It is recognized for its high yield and impressive nutritional quality. The oil extracted from peach palm pulp can serve as an intermediate source of omega-6 fatty acids (4.9–8.6%). In addition, it also contains substantial proportions of oleic, linoleic, and linolenic acids, which are known for their cardiovascular benefits and oxidative stability. Refs. [4,15] show a clear move away from old solvent-based extraction methods toward cleaner, safer technologies for obtaining peach palm oil, and [45] also supported the transition to green extraction processes that meet the growing demand for clean label, sustainable ingredients while maintaining oil quality. Among these processes, ultrasound-assisted extraction combined with ethanol achieved oil yields of up to 8.9% while preserving key bioactive constituents like carotenoids and unsaturated fatty acids, particularly in red and yellow peach palm fruits. However, peach palm oil has been reported to be relatively susceptible to oxidation, likely due to its high omega-9 (oleic acid) content. Fatty acids are inherently prone to oxidative degradation because of the double bonds in their molecular structure, leading to free radical chain reactions that can compromise both the nutritional and the sensory quality of the oil. In parallel, supercritical CO_2_ extraction enables the recovery of high-purity oils with negligible solvent residues, making them ideal for health-oriented applications. These lipids, naturally rich in oleic, linoleic, and linolenic acids, are prized for their heart health benefits and resistance to oxidation, fitting neatly with the growing demand for nutritious, sustainable, plant-based foods [4,45]. Despite this, Soares et al. [4] also documented the antimicrobial characteristic of oil extracted using hexane from peach palm bark against strains of *Staphylococcus aureus* 24 h after the addition of 10 µL oil. Given the growing interest in peach palm as a potential sustainable lipid source for food applications, the fatty acid profile of peach palm pulp lipid extracts is summarized in Table 3. This profile highlights the relative proportions of saturated and unsaturated fatty acids and provides compositional information relevant to nutritional quality and oxidative stability.

(b) Carotenoid concentration for natural coloring

Among the fat-soluble constituents of peach palm, carotenoids are also considered important bioactive nutrients and are present in high concentrations in the fruit. The intensely colored carotenoid compounds from peach palm constitute valuable natural colorants and bioactive substances. Various studies have found that yellow, orange, and red peach palms are rich in carotenoids, and the stage of fruit maturation strongly influences their total carotenoid content. Moreover, fresh fruits of the orange peach palm variety exhibit the highest total carotenoid content, followed by the red and yellow cultivars, demonstrating a clear relationship between color intensity and carotenoid concentration.

Spacki et al. [15] demonstrated that carotenoid-rich fractions from peach palm can be effectively extracted using ultrasound-assisted extraction (UAE), ionic liquid systems, and supercritical CO_2_ methods with recyclable, low-toxicity solvents. In addition, ref. [4,46] reported that the red and yellow cultivars possess the highest carotenoid concentrations, underscoring their potential in clean label product development and functional food formulation.

Carotenoids are subject to instability, which can be influenced by their chemical composition (carotene or xanthophyll), molecular structural configuration (cis or trans), esterification, and the cellular matrix, as well as by processing and storage conditions. Notably, higher contents of total carotenoids after cooking have been reported in peach palm fruit compared with fresh samples, possibly due to the release of carotenoids from cell walls. This thermal effect may also lead to the formation of isomers such as Z-β-carotene, Z-γ-carotene, and Z-lycopene, which have been identified in peach palm fruit and may contribute to variations in bioactivity. Furthermore, the bioaccessibility of carotenoids from peach palm can be enhanced by incorporating them into lipid-based matrices. For instance, carotenoids extracted from peach palm pulp by ultrasound and added to a mayonnaise emulsion were found to be 11 times more bioaccessible after in vitro digestion compared with those in freeze-dried fruit. The utilization of peel fractions also supports natural pigment recovery and promotes sustainability in ingredient procurement [4,15,45].

(c) Antioxidant activity of peach palm extracts

Lipid and carotenoid-rich extracts from peach palm exhibit notable antioxidant activity resulting from their polyphenol, tocopherol, and phytosterol contents. Various extraction methods such as ultrasound and microwave assistance have been demonstrated to improve the extraction efficiency of phenols and antioxidant potential, exceeding the performance of traditional methods. These bioactive extracts contribute to functional food and nutraceutical development, corresponding with present-day demands for health-beneficial, sustainable ingredient sources [4,15]. An overview of advanced extraction technologies available for lipid and bioactive compound recovery from peach palm, including their process parameters, compositional highlights, and functional applications, is presented in Table 4. Unless otherwise stated, quantitative values are reported on the basis used in the original studies (fresh weight, dry weight, or extract basis), which is explicitly indicated to avoid misinterpretation.

Current advancements in extraction technology enable zero-waste processing approaches and circular ingredient development for the sustainable food industry, exploiting lipids and carotenoids from complete peach palm biomass fractions for innovative plant-based food applications.

**Figure 4:**
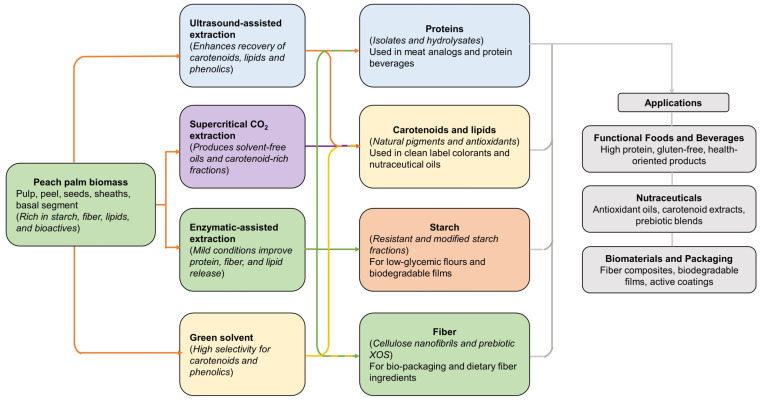
Modern extraction technologies for peach palm; from biomass to functional ingredients.

Figure 4 shows an integrated schematic of modern extraction technologies for peach palm, outlining how different green methods yield functional ingredients for food, nutraceuticals and biomaterial applications. Current advancements in extraction technology enable zero-waste processing approaches and circular ingredient development for the sustainable food industry, exploiting lipids and carotenoids from complete peach palm biomass fractions for innovative plant-based food applications.

(d) Bioactive compounds identified in peach palm and their biological activities

Several studies have identified diverse classes of bioactive compounds in peach palm, including carotenoids, phenolic compounds, tocopherols, phytosterols, and organic acids. These compounds contribute to antioxidant, anti-inflammatory, antimicrobial, and cardioprotective activities, supporting the functional food potential of peach palm. Table 5 summarizes the main bioactive compounds reported in different peach palm fractions, together with their biological activities and supporting references.

### 2.4. Integrated Processing Approaches

(a) Zero-waste processing concepts

Although the concept of “zero-waste” is frequently used to describe fully integrated biorefinery and valorization strategies, the complete elimination of waste is rarely achievable in practice due to unavoidable material losses, process residues, and energy demands. In the case of peach palm, a near zero-waste approach can nevertheless be pursued through the sequential utilization of different plant fractions. Edible pulp can be directed to food applications, while peels, sheaths, and basal portions can be converted into dietary fiber concentrates, carotenoid-rich extracts, fermentable substrates, or biopolymer precursors. Residual solids may further be valorized through fermentation or energy recovery processes. This cascading use of biomass reduces disposal streams and improves resource efficiency, even if true zero-waste conditions remain theoretical rather than absolute [6,15].

(b) By-product valorization strategies

A wide range of peach palm by-products, including peels, inner sheaths, and stem segments, are currently acknowledged as abundant sources of dietary fiber, xylans (for prebiotic xylooligosaccharide production), cellulose (for nanofibril applications), phenolic compounds, and carotenoids [42]. Principal valorization approaches include the production of high-fiber flours for the application as functional food ingredients and emulsifying agents; extraction of natural pigments and antioxidant compounds, supporting food and nutraceutical sectors; alkaline or enzymatic hydrolysis of xylans for xylooligosaccharide (prebiotic) generation, producing antioxidant-rich fractions; and extraction with nanoprocessing of cellulose for bio-based packaging materials or rheological modifiers. Through proper process integration and sequencing, these methodologies reduce disposal expenses and establish new revenue opportunities for processing facilities and rural farming communities [15].

(c) Multi-component extraction systems

Recent progress in multi-component extraction have made it possible to isolate lipids, carotenoids, dietary fibers, starch, and phenolic compounds, either sequentially or simultaneously, from both edible and inedible parts of peach palm. Using techniques such as ultrasound-assisted extraction, supercritical CO_2_, and ionic liquid-based extraction enables the targeted recovery of specific compounds without significant loss in biological activity. These compounds can be used in food, cosmetics, pharmaceutical, and biomaterial applications [4,14,15]. When combined with enzymatic or fermentation-based processes, for instance, fermentation using Trichoderma or Lentinula, these systems generate additional value through enzyme production (amylases, xylanases) and prebiotic oligosaccharide synthesis. [15].

For instance, Lima et al. [47] studied the performance of *B. gasipaes* residues as a substrate for the growth of a mycelium-based composite on *Lentinula edodes*. The composite formed displayed close values compared to other mycelium-based composites on compressive strength and elastic modulus. The authors concluded that pupunha residues are a potential alternative for mycelium-based composites. The production and commercialization of mushrooms (healthy functional foods) using peach palm residues could result in socio-environmental benefits by increasing the income of the involved individuals and by reducing environmental liability. Therefore, developing sustainable, integrated biorefinery models is essential for the full exploitation of peach palm resources. Such approaches not only minimize environmental impact but also enhance economic potential, positioning peach palm residues as key contributors to future zero-waste and circular bioeconomy systems. Table 6 summarizes the specialized valorization strategies for various peach palm by-products, highlighting the functional materials generated and their role in the circular bioeconomy. Sustainable, integrated biorefinery strategies are therefore critical for the comprehensive exploitation of peach palm resources, corresponding with global movements towards zero-waste systems and circular bioeconomy advancement. Such frameworks address environmental liabilities while enhancing economic value, establishing peach palm residues as essential resources in future food and bioproduct supply chains.

## 3. Valorization of By-Products and Circular Uses

**Figure 5:**
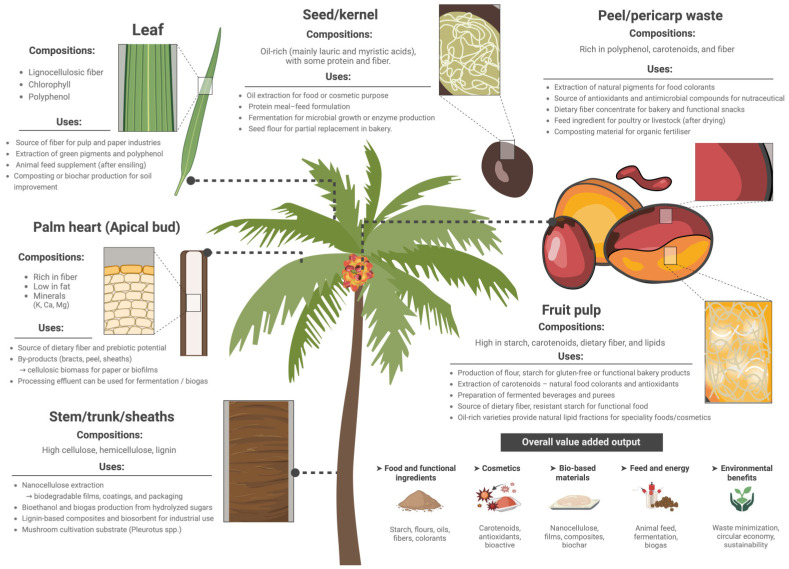
Valorization potential of peach palm parts and derived outputs. https://BioRender.com/v81vyht. Accessed on 5 February 2026.

The valorization potential of different anatomical parts of peach palm, detailing their composition, principal uses, and value-added outputs within a circular bioeconomy framework, is shown in Figure 5. The production and consumption of peach palm generates a huge volume of by-products [48]. The utilization of processing by-products represents a key strategy for achieving the circular biomass management of peach palm within sustainable food systems. During peach palm extraction, it is estimated that approximately 84% of the total weight of the palm is wasted. Nevertheless, chemical analysis of these fractions reveals high dietary fiber contents ranging from 59% to 68%, alongside protein levels of 8–12%, confirming their potential as sustainable raw materials for high-fiber or protein-enriched functional ingredients [6,15].

In addition to the macronutrient composition of these by products, these so-called wastes (by-products) are also rich in nutraceutical compounds such as myo-inositol—a bioactive compound with documented metabolic and prebiotic functions—and a spectrum of organic acids that contribute to potential antioxidant and antimicrobial activities. Recent progress in extraction and processing has made it possible to transform the sheath and basal fractions into marketable products, including high-fiber flours and dietary supplements, xylooligosaccharide-based prebiotic formulations, and cellulose nanofibrils suitable for use in packaging materials or as food texture modifiers [6,15].

A number of valorization pathways have been established within circular economy frameworks, encompassing enzymatic hydrolysis for xylooligosaccharide production, solid-state fermentation for edible mushroom cultivation or enzyme biosynthesis, and nanoprocessing techniques designed to improve cellulose recovery from processing residues [54]. Implementing these strategies not only reduces environmental burden but also opens new avenues for ingredient innovation and economic benefits for processors and local producers [6,15].

In conclusion, the external and internal sheaths together with the basal segment of peach palm should no longer be considered waste but should be recognized as valuable co-products with transformative potential for the development of fiber- and protein-rich foods, nutraceutical formulations, and bio-based materials within modern sustainable food systems. A comparative overview of the valorization pathways for major peach palm by-product is provided in Table 7.

### 3.1. Potential Uses of Peach Palm By-Products

(a) Dietary fiber ingredients

Peach palm residues, particularly those derived from the median sheaths and stem portions, are rich in non-starch polysaccharides and can be processed into fiber-enriched flours suitable for improving the nutritional profile of cereals, baked products, and similar food matrices [4,13]. The fractions of these flours predominantly consist of insoluble dietary fibers, known to support intestinal function, aid in cholesterol regulation, and contribute to glycemic control when consumed regularly. In addition, the flour obtained from pulp and peel retains substantial amounts of total dietary fiber and bioactive compounds. This extends the potential of peach palm as functional ingredients across a range of modern food formulations [15].

(b) Functional food additives

The by-products of peach palm after processing can be used as food additive materials. The one such example includes the derived flour from peach palm. The flour exhibits remarkable water and oil absorption capacities, emulsifying ability, and structural stability properties. Due to these unique properties of flour, it can be employed as an effective natural emulsifier or textural modifier. It can also be used as the matrix for encapsulating bioactive molecules in baked goods, dairy formulations, and meat product applications [52]. In addition, the antioxidant properties and carotenoid concentrations in these by-products can often be used as natural coloring agents and provitamin A reservoirs. This contributes to nutritional value augmentation and conformity with clean label consumer preferences [4,13,15,45].

(c) Mushroom substrates

Lignocellulosic materials such as leaf sheath and middle sheath components derived from peach palm act as suitable substrates for solid-state fermentation and edible or medicinal mushroom production systems [15]. Camilleri et al. [55] reported that peach palm residues create an optimal environment for fungal growth, particularly for *Lentinula edodes* and *Pleurotus ostreatus*, resulting in mycelium-based composites with notable structural strength and nutritional value. In parallel, findings from [13,15] highlighted the potential of fermentation-derived by-products as functional feed components that influence glycemic and lipid metabolism, as well as promising precursors for developing sustainable mycelium-based biomaterials [13,15]. As per [50], others evaluated *P. ostreatus* yield in peach palm leaves supplemented with rice bran and reached yields between 20.6 and 42.3% (*w*/*w*). They recorded the protein content of 24.1 g/100 g in *P. ostreatus* produced in peach palm leaves. The protein content is one of the desirable and most important parameters in mushrooms, especially when used in plant-based diets, due to the restrictions in the consumption of sources of proteins from animal origin [50].

(d) Biomaterials

By-products from peach palm subjected to upcycling processes are gaining recognition for their applicability in biomaterial development and environmentally sustainable chemistry. Recent developments indicate that the peach palm’s lignocellulosic wastes hold great potential for being upcycled into valuable biotechnological products such as prebiotics, enzymes, cellulose and high fiber flours. Combination of chemical and mechanical treatments of peach palm sheaths led to the production of cellulose nanofibrils. Martins et al. [49] used these cellulose nanofibrils to improve the characteristics of cassava starch films. It was observed that physical reinforcement was the main effect observed in cassava starch films containing cellulose nanofibrils according to the analysis of mechanical strength and permeability. The spectroscopic data further revealed a possible formation of crosslinking between starch and cellulose nanofibrils, which can positively influence the tensile strength of such films. Additionally, starch extracted from non-traditional sources, including white peach palm varieties, displays distinctive functional characteristics and pasting behavior profiles, thereby broadening utilization prospects in biodegradable film technologies, pharmaceutical delivery systems, and as gel-forming or binding agent alternatives [4,13,15].

(e) Biorefinery resources

Peach palm by-products are rich in lignocellulosic materials, mainly cellulose, hemicellulose, and lignin, which makes them suitable feedstocks for biorefinery-based processing systems. In a biorefinery context, biomass is fractionated into multiple value-added streams rather than treated as waste, allowing the sequential conversion of structural polysaccharides and associated compounds into food ingredients, chemicals, and energy carriers.

The effective application of this concept requires consideration of the macromolecular organization of peach palm tissues, in which cellulose and hemicellulose are embedded within lignin-rich matrices that limit enzymatic accessibility. Accordingly, fungal pretreatment has been reported to enhance enzymatic hydrolysis efficiency by partially disrupting these structural associations, thereby enabling the release of fermentable sugars for the production of second-generation bioethanol, organic acids, and other renewable chemicals [56].

Spacki et al. [6,15] and Soares et al. [13] further demonstrated that xylan derivatives obtained from the inner sheath can be converted into prebiotic xylooligosaccharides. Following carbohydrate recovery, the remaining fiber-rich residues may be directed toward energy recovery processes or used as feedstock for bio-based materials. Such a cascading utilization of peach palm fractions illustrates how understanding macromolecular connectivity can guide the selection of appropriate processing routes within a biorefinery framework. These integrated pathways support circular bioeconomy strategies by linking agricultural residues with value-added production systems. Table 8 provides a comparative overview of commercial and research-stage peach palm products applied in plant-based foods.

### 3.2. Commercial Perspectives of Peach Palm Valorization

Currently, the commercial exploitation of peach palm is mainly focused on edible fruits and heart-of-palm products, while most bioactive and functional ingredients derived from by-products remain at the research or pilot scale. Dietary fiber concentrates, starch-based ingredients, and carotenoid-rich extracts represent the most promising product categories for food and nutraceutical applications. Among the processing technologies discussed, conventional solvent extraction and mechanical pressing remain the most widely used methods at the industrial scale due to their operational simplicity, lower capital cost, and established regulatory acceptance. Emerging techniques such as ultrasound-assisted, enzymatic, and supercritical carbon dioxide extraction have demonstrated superior selectivity and product quality; however, their commercial adoption is still limited by equipment costs, scale-up constraints, and energy requirements. Nevertheless, increasing demand for clean label, sustainable, and plant-based ingredients is driving interest in greener extraction and fractionation technologies, suggesting that peach palm by-products could progressively transition from laboratory-scale valorization to industrial biorefinery-based applications.

## 4. Challenges and Research Needed

### 4.1. Toxicity Issues

The raw consumption of peach palm fruit is limited by the presence of calcium oxalate crystals, which induce irritation and necessitate prior processing. The sharp, needle-like crystals are concentrated in the pulp as well in the peel of peach palm fruit. When consumed raw, these crystals cause an immediate burning sensation, irritation, and pain in mouth or throat [15]. Soares et al. [4] documented that even small amounts of uncooked fruit can trigger airway swelling and, in severe cases, respiratory distress. Moreover, long-term or high-level exposure to these crystals may also affect kidney and liver health, underscoring the importance of proper processing before consumption [5].

Heat treatment, either cooking or boiling, serves as an effective way to make peach palm edible without any harmful effect [13]. Thermal processing not only solubilizes these crystals but also deactivates antinutritional and irritants present in the fruit. Santos et al. [59] claimed that cooking the fruit at 105 °C for 20 min eliminates these oxalate crystals from the peel. In addition, cooking also inhibits antinutritional factors such as trypsin inhibitors, inactivating peroxidase enzymes present in the pulp. These components can otherwise irritate the throat mucosa. Overall, it improves the safety and flavor profile of the fruit. In Amazonian cuisines, the fruits are often boiled in salted water. This enhances the taste while eliminating toxic elements, thereby providing complementary benefits. This essential step preserves the fruit’s valuable nutrients, including fiber, vitamins, and bioactive phytochemicals [4,5,13,15].

Despite the proven effectiveness of thermal processing in enhancing the safety of peach palm fruit, important research gaps persist. Optimized thermal parameters for cooking are still required to achieve complete detoxification without compromising nutritional or sensorial properties. As peach palm has emerged beyond its native regions as a plant-based food ingredient, there is a need to ensure safety and consistency under industrial scale processing conditions. Moreover, improved methods for detecting and quantifying calcium oxalate residues are necessary to support both regulation and quality control. Addressing these gaps will enhance consumer safety and help establish peach palm as a reliable, sustainable ingredient in future food innovations [15].

### 4.2. Nutritional Variability

There is variability in the nutritional values that can be explained by the different *B. gasipaes* varieties, size, and starch oil composition of the fruit [4,5]. The fruit also varies genetically and from region to region. The major macronutrients vary significantly among accessions [13]. Detailed discussions on nutritional composition, fatty acid profiles, and bioactive compounds have been comprehensively addressed in our previous review (‘Unlocking the potential of peach palm for plant-based foods’) and are therefore only briefly summarized here. For instance, peach palm is known for its high starch, fiber and carotenoid content with modest protein levels when compared with most cereal grains. In certain processed forms, such as flours from albino varieties or certain industrial by-products, protein content can increase to 16–20 g per 100 g (dry basis), though such cases are uncommon.

The variations in nutritional profiles among the varieties, particularly pigmented and albino varieties, present novel opportunities for selective breeding among the varieties. This will result in enhanced protein quality and overall nutritional value for specific food applications or population needs. Unlocking the full potential of peach palm will depend on detailed genetic studies, improved protein isolation methods, and thoughtful formulation strategies to achieve optimal amino acid balance in next-generation plant-based food systems [4,5].

### 4.3. Functional Limitation

The functional properties of peach palm are limited, majorly in terms of gelling and foaming ability. These properties restrict its direct use in products that rely on viscosity control, emulsification, or stable foam structures. The high gelatinization temperature, low oil absorption and emulsifying capacity of peach palm requires blending with certain other flours or use of certain modification methods (as detailed above) to improve texture and performance in bakery or processed food applications [4,5].

### 4.4. Scalability Barriers

The large-scale commercial utilization of peach palm as a food component is limited due to a number of factors, such as processing difficulties, sensory challenges, evolving regulations for novel plant proteins, etc. As stated earlier, optimized and effective processing is still required to reduce toxicity without compromising the nutritional and sensorial properties. Because peach palm is considered an emerging protein source, manufacturers may also face regulatory hurdles that demand thorough safety assessments, toxicological studies, and detailed compliance documentation before products can enter the market [15,60,61]. Table 9 summarizes the key scalability barriers and mitigation strategies that currently limit the large-scale valorization of peach palm in sustainable food systems.

### 4.5. In Silico Approaches for Bioactivity Prediction

In silico techniques, including molecular docking, quantitative structure–activity relationship (QSAR) modeling, and bioactivity prediction platforms, are increasingly used to elucidate the mechanistic interactions between food-derived bioactive compounds and biological targets. For peach palm, such computational tools could be applied to predict the binding affinity of carotenoids, phenolic acids, and phytosterols to key molecular targets involved in oxidative stress, inflammation, and lipid metabolism. These approaches may assist in prioritizing compounds for further in vitro and in vivo validation, reducing experimental cost and time while improving mechanistic understanding. Integration of computational modeling with experimental extraction and characterization data could therefore accelerate the development of peach palm-based functional ingredients and nutraceutical formulations.

## 5. Conclusions

Peach palm is emerging as a nutritionally rich crop with strong adaptability. Some research done on it states that it has adapted well to modern extraction and processing methods. Its extremely rich and diverse composition enables to produce the starches with desirable functional properties, i.e., lipid extracts rich in bioactive compounds and high-quality protein isolates. Such versatility positions peach palm as a promising raw material for the development of next-generation plant-based foods. Valorization of the entire plant, including the peel, pulp, and processing residues, not only enhances resource efficiency but also aligns with circular bioeconomy principles aimed at achieving zero-waste production. With recent advancements in sustainable extraction and green processing methods, the quality and commercial potential of peach palm-derived ingredients has improved considerably. Continued interdisciplinary research focusing on process optimization, functionality, and product innovation will further establish peach palm as a sustainable, high-value crop. Its integration into food, nutraceutical, and biomaterial applications reflects a step toward a more resource-efficient and environmentally responsible food system.

## Figures and Tables

**Table 1:** Processing methods for peach palm and their plant-based food applications.

Methods	Parameters	Targeted Component	Key Quality/Yield (Basis as Reported)	Food Applications	Advantages
Traditional Boiling/Cooking	Boil whole fruit (20–60 min), peel, manual pulp separation	Pulp, cooked fruit	Reduced oxalate, increased starch	Direct eating, flour, puree	Inactivates antinutrients, ready for gluten-free use
Sun or Forced-Air Drying	Dry cooked pulp or flour at 50–60 °C	Flour, pulp	Lower water activity, stable	Baking, bread, extrudates	Extended shelf life, stable color/nutrients
Milling/Grinding	Mechanical grinding, post-cooking and drying, sieving (particle size control)	Flour (pulp/peel), starch	Gluten-free, fiber-rich flour	Gluten-free bakery, cookies, extruded snacks	Different mesh size for texture/functionality
Traditional/Conventional Oil Extraction	Solvent-based (hexane, ether) or mechanical pressing	Oil (pulp/seed), carotenoids	7–21% *w*/*w* (oil yield, basis as reported)	Spreads, enriched foods, functional oils	High vitamin A, tocopherols, phytosterols
Ultrasound-Assisted Extraction (UAE)	Ethanol as solvent, 30 min, 20 kHz, 50 °C, variety-dependent	Lipids, carotenoids, polyunsaturates	2–8% (variety), enriches carotene	Nutraceutical oils, colorants, PUFA for health	“Green”, less solvent, higher antioxidant retention
Supercritical CO_2_ Extraction	Supercritical CO_2_ at 300 bar, 40 °C, pulped fruit or peel	Carotenoids, oils	Max carotenoid, low solvent residue	Functional/therapeutic food colors/ingredients	Clean, scalable, preserves labile compounds
Ionic Liquid Extraction	Imidazolium-based IL, recyclable solvent, 30–60 min, controlled recycle	Carotenoids, phenolics	172 μg/g, 94% IL recovery	Emulsified food, supplements	High yield, greener, repeated use
Starch Extraction and Fractionation	Water or acid extraction, sieving, separation of granules	Amylose, amylopectin, resistant starch	55–72%, 14–20% amylose	Low glycemic breads, porridge, gluten-free flour	Produces slow-digesting starch for metabolic health
Enzymatic/Physical Protein Extraction	Solubilization and precipitation post-cooking, mechanical pressing	Protein concentrate/isolate	Up to 4–7% protein in flour	Fortified flours, alternative protein foods	Functionality as foaming/emulsifying agent
Modern Gluten-Free Flour Production	Combined cooking, drying (forced air or freeze-drying), fine milling	Whole flour, composite ingredients	High water- and oil-binding capacities	Gluten-free cakes, bread, pasta, batters	Provides structure, color, fiber, minerals
Extrusion Cooking	High-temp, short-time, peach palm/corn blends	Texturized flour, enriched extrudate	Maintains pigments, texture control	Cereal, breakfast, snack extrudates	Enhances carotenoid retention, fiber enrichment
Microwave-Assisted Extraction	Use with solvent (e.g., ethanol), short bursts for extraction of bioactives	Carotenoids, phenolics	Higher yields, darker color oil	Functional oils, provitamin A concentrates	Efficient, less time, gentle for sensitive compounds
Enzyme Production (from Waste/Peel)	Solid-state fermentation with Trichoderma or Pleurotus spp., supplement N-source	Amylase, hydrolytic enzymes	29–53 U/g (amylase, optimized mix)	Starch hydrolysis in baking/foods, fermentation	Waste reduction and creates value for by-products
Color and Phytochemical Extraction	Sequential solvent extraction, use of green solvents	Flavonoids, tocopherols, carotenoids	357 mg/kg oil for total carotenoids	Natural colorants, antioxidant-rich foods	“Green” chemistry, for pigment/nutrient-rich foods
Formulation in Plant-Based Foods	Blending with other cereal or legume flours, texturization, color stabilization	Final formulated ingredient	High sensory acceptance (>70%)	Cookies, breads, vegan spreads, health snacks	Increases micronutrient density, functional fibers

Note: Percentage values refer to yields as reported in the original studies (e.g., based on fresh material, dry material, or oil fraction). Source: [4,6,7,13,14,15].

**Table 2:** Recent advances in protein extraction, fractionation, and functional modification from peach palm and plant sources.

Extraction Method	Source	Protein Content (%, Basis as Reported)	Modification Steps	Functional Traits Developed	Food Applications
Hydrothermal extraction/milling	Cooked pulp, flour	1.8–4.6	Cooking, milling, sieving	Increased solubility, partial denaturation	Gluten-free bakery, breads, snack powders
Dry fractionation	Dried flour	Up to 17–20	Fine grinding, dry sieving	Improved water/oil holding, emulsification	Cakes, biscuits, extruded cereals
Alkaline/aqueous protein extraction	Pulp, flour	8–25 (concentrate)	Homogenization, aqueous or alkali leaching, centrifuge	Higher protein purity, reduced antinutrients	Protein concentrates, supplement blends
Enzymatic assisted modification	Pulp or flour	Variable	Protease-assisted extraction, hydrolysis	Enhanced solubility, antioxidant peptides	Functional protein isolate, protein beverages
Ultrasound-assisted extraction	Pulp, flour	Up to 25	Acoustic-assisted aqueous extraction	Improved yield and foaming	Protein enrichment in bakery, snacks
Isoelectric precipitation	Aqueous extract	40–60 (isolate)	pH shift, protein separation, drying	Concentrated fractions, tailored gelling	Vegan cheese, meat analogues
Functional protein modification	Protein flour/extract	Application-specific (variable)	Enzymatic or heat-induced changes, blending	Water and oil binding, improved emulsification	Texturizers, beverage stabilizers

Note: Protein contents are reported as provided in the cited studies and may be expressed on a fresh-weight (fw), dry-weight (dw), or isolate basis. Source: [4,5,7,13,14,15,16,30,31,32,33,34].

**Table 3:** Fatty acid composition of peach palm pulp lipid extract obtained by ultrasound-assisted extraction.

Fatty Acid (% Total FA)	Red	Yellow	Green	White
Lauric (C12:0)	0.01 ± 0.00	0.02 ± 0.00	0.01 ± 0.00	0.01 ± 0.00
Myristic (C14:0)	0.08 ± 0.00	0.15 ± 0.00	0.08 ± 0.00	0.10 ± 0.00
Palmitic (C16:0)	23.77 ± 0.15	28.96 ± 0.23	33.86 ± 0.34	42.62 ± 0.43
Stearic (C18:0)	Nd	0.70 ± 0.02	Nd	1.87 ± 0.23
Arachidic (C20:0)	0.14 ± 0.02	0.10 ± 0.00	0.12 ± 0.03	0.19 ± 0.00
Palmitoleic (C16:1)	9.89 ± 0.34	13.23 ± 0.12	3.98 ± 0.91	4.99 ± 0.03
Oleic (C18:1n−9)	60.20 ± 0.50	44.85 ± 0.41	57.62 ± 0.14	40.73 ± 0.54
Linoleic (C18:2n−6)	4.04 ± 0.61	8.05 ± 0.91	2.03 ± 0.14	6.95 ± 0.17
α-Linolenic (C18:3n−3)	1.48 ± 0.24	2.50 ± 0.07	0.54 ± 0.23	2.14 ± 0.34

Note: Values are expressed as percentage of total fatty acids as reported in the original study. Nd= Not detected. Source: [45].

**Table 4:** Overview of advanced extraction technologies for lipid and bioactive compound recovery from peach palm.

Extraction Method	Solvent Type	Main Compound	Extraction Yield (%, as Reported in Original Studies)	Highlights	Functional Properties	Applications	Disadvantages	Reference
Ultrasound-Assisted Extraction	Ethanol	Carotenoids, Lipids	Up to 8.9 (red var.)	High beta-carotene, unsat. FA	Preserves thermolabile pigments	Clean label oil, natural pigment	Limited scalability; possible oxidation if ultrasound intensity is not controlled; higher equipment cost than conventional extraction	[8,45]
Supercritical CO_2_ Extraction	CO_2_	Carotenoids, Lipids	6.1–8.2	Strong antioxidant retention	Solvent-free, scalable	Functional oil, nutraceuticals	High capital and operating cost; requires high pressure; low efficiency for polar compounds without co-solvent	[11]
Ionic Liquid Extraction	Ionic liquids	Carotenoids, Phenolics	Up to 172 µg/g extract	Enables selectivity	High recyclability, green tech	High-value pigment fractions	Solvent recovery required; regulatory acceptance for food use is limited; potential toxicity depending on ionic liquid type	[10]
Microwave-Assisted Extraction	Ethanol/Water	Lipids, Bioactives	6–10	Moderate yield, rapid process	Retains bioactivity	Pigment/antioxidant extracts	Risk of uneven heating; possible degradation of heat-sensitive compounds; scale-up challenges	[15]
Enzymatic-Assisted Extraction	Enzyme-buffer	Phenolics, Lipids	5–9	Increases extractability	Mild conditions, low residue	Antioxidant/fat ingredient	High enzyme cost; long processing time; sensitive to pH and temperature variations	[6,9]
Conventional Solvent	Hexane/Ether	Lipids	7–21	High yield, less selectivity	Food-grade restrictions	Bulk oil ingredient	Use of toxic/flammable solvents; environmental burden; solvent residues; low selectivity	[4,18]
[18] Mechanical Pressing	—	Lipids	3–8	Preserves natural composition	Low yield, safe process	Whole oil, unrefined ingredient	Low extraction efficiency; requires pretreatment; not suitable for bound lipids	[18]
Solid-State Fermentation	—	Phenolics, Bioactives	Variable	Generates novel bioactives	Nutritional and functional	Enriched flours, extracts	Long processing time; contamination risk; batch-to-batch variability; difficult process control	[6]

Note: Data are reported as provided in the original studies. Percentage values refer to extraction yield relative to the initial material or oil fraction, as specified in the cited sources.

**Table 5:** Bioactive compounds identified in peach palm and reported biological activities.

Compound Class	Identified Compounds	Plant Fraction	Reported Biological Activity
Carotenoids	β-carotene, α-carotene, lutein, zeaxanthin, lycopene	Fruit pulp, peel	Antioxidant, provitamin A activity, immune modulation
Phenolic compounds	Gallic acid, ferulic acid, caffeic acid, chlorogenic acid	Peel, pulp, sheath	Antioxidant, anti-inflammatory
Tocopherols	α-tocopherol, γ-tocopherol	Fruit oil	Lipid oxidation inhibition, cardiovascular protection
Phytosterols	β-sitosterol, campesterol, stigmasterol	Fruit oil, seed	Cholesterol-lowering, anti-inflammatory
Organic acids	Citric acid, malic acid, succinic acid	Pulp, peel	Antimicrobial, pH regulation, antioxidant synergy
Polysaccharides	Pectin, hemicellulose, resistant starch	Pulp, by-products	Prebiotic, gut microbiota modulation
Myo-inositol	Myo-inositol	Sheath, basal portion	Metabolic regulation, prebiotic effect

Source: [4,7,9,10,11,15,42,43,44,45].

**Table 6:** Specialized valorization strategies for peach palm by-products; functional materials and circular bioeconomy innovations.

By-Product Fraction	Main Component	Processing Technology	Function	Application	Innovation	Limitations	Reference
External Sheath	Dietary fiber (cellulose, hemicellulose), low protein	Alkaline/enzymatic extraction, hydrothermal treatment	XOS (xylo-oligosaccharides), cellulose nanofibrils	Prebiotic blends, biopolymer composite materials	Edible packaging, XOS-based gut health supplement	Requires chemical pretreatment; generation of alkaline wastewater; variability in fiber composition depending on maturity	[14,48,49]
Internal Sheath	Complex fiber, pectin, trace minerals	Enzyme refinement, composting, microbial fermentation	Natural pectin fraction, biofertilizer	Functional hydrocolloid, soil amendment	Microbial valorization for organic farming	Low pectin yield compared with citrus sources; microbial processes require strict control; slow processing rate	[14,42,50]
Basal Segment	Lignocellulose, residual carbohydrate	Saccharification, anaerobic digestion, drying	Biogas, resistant starch flour	Renewable energy, specialty feeds	Circular plant energy, starch for clean label baking	High lignin content limits enzymatic hydrolysis; requires energy-intensive pretreatment; limited food-grade applications	[6,35,48]
Fruit Residue	Pulp fiber, polyphenols, organic acids	Aqueous extraction, enzyme hydrolysis, drying	Polyphenol-rich colorant, citric extracts	Functional colorant, natural acidulant	Active packaging ingredient for shelf-life extension	Polyphenols are sensitive to heat and oxidation; aqueous extracts are dilute and require concentration; seasonal variability	[7,51,52]
Seed/Kernel Waste	Oil, lignin, bioavailable micronutrients	Cold pressing, pyrolysis, ultrafiltration	Bio-oil, micronutrient concentrate	Biolubricant/green solvent, fortificant	Micronutrient delivery for new nutraceuticals	Low oil yield by pressing; pyrolysis products not suitable for food use; additional refining required for edible applications	[18,53]

**Table 7:** Comparative overview of valorization pathways for major peach palm by-product fractions; composition, key nutraceuticals, functional applications and recent trends.

By-Product	Fraction of Biomass	Fiber (%)	Protein (%)	Key Nutraceuticals	Advanced Valorization Products	Applications	Recent Trends	Disadvantages	Reference
External sheath	~83.6	59–68	8–12	Myo-inositol, organic acids, polyphenols	Fibrous flour, cellulose nanofibrils, polyphenol-rich extracts	Dietary fiber supplements, biodegradable packaging materials	Global growth in bio-based packaging; fiber used in active packaging	High fiber content can reduce palatability; requires particle-size control and pretreatment; possible contamination if poorly handled	[15,42,49]
Internal sheath	~83.6	59–68	8–12	Xylooligosaccharides, myo-inositol	XOS, bioactive-rich flour, bioplastic precursors	Prebiotics, functional food fortification, bioplastics	XOS gaining market share as functional ingredient	XOS production requires controlled hydrolysis; possible bitter taste at high concentrations; processing cost	[15,48,49]
Basal portion	~83.6	59–68	8–12	N-acetyl-D-glucosamine, amino acids	Substrate for fermentation, food hydrocolloid source	Feedstocks, enzyme production, food thickeners	New bioprocessing for cell-cultured meat media	Composition varies with plant maturity; fermentation efficiency depends on pretreatment; limited direct food applications	[6,15,50]

**Table 8:** Comparison of major peach palm-based products for plant-based food applications.

Product Type	Nutritional Profile	Key Functional Properties	Example Foods	Consumer/Market Relevance	Sustainability/Valorization Aspects
Peel Flour	13–14% lipids, ~6% protein, ~62% carbohydrates, high fiber	Oil/water binding, texture, fiber	Gluten-free bread, cakes, snacks	Celiac/health segment	Produced from waste streams, valorizes by-products
Protein Isolate	40–60% protein, all essential AAs	Foaming, emulsifying, texture	Alt meat, dairy analogs, protein bars	Premium/high-protein seekers	Low amylose/high amylopectin aids processing, sustainable source
Lipid Extracts	High USFA (up to 70%), ω-3, ω-6, β-carotene up to 748 µg/100 g extract	Antioxidant, cardiovascular function	Margarine, supplements, enriched drinks	Heart health, wellness focus	Green extraction, edible oil from by-products
Colorant Extract	Carotenoids up to 172 µg/g (peels), up to 748 µg/100 g in oil	Natural pigment, antioxidant	Coconut drinks, baked goods	Clean label, fortification	Utilization of epicarp waste, green solvent extraction
Starch Flour	68–79% starch, low protein, gluten-free	Binder, texture, gelation	Cookies, cakes, meat product fillers	Processed food innovation	Seasonal valorization, replaces wheat for GF options

Source: [4,10,45,52,57,58].

**Table 9:** Key scalability barriers and mitigation strategies for peach palm valorization in sustainable food systems.

Barrier Type	Description/Example	Impact on Scale-Up	Potential Solutions/Strategies
Genetic and Raw Material Variability	Limited development of high-yielding, high-quality varieties; genetic erosion; fragmented wild populations	Inconsistent product quality	Breeding programs, in vitro culture protocols, conservation
Agro-Industrial Waste Management	80–90% of the palm mass is by-product; waste disposal challenges impede processing scale-up	Increased costs and complexity	Circular economy valorization, upcycling, fiber bioproducts
Processing Technology Limitations	Slow adoption of green extraction, enzymatic modification, and waste bioproduct tech; high energy/water use	Low efficiency, high costs	Biotechnological pretreatment, solid-state fermentation
Market and Supply Chain Fragmentation	Multi-stakeholder chains, price fluctuations, long farm-to-market chains	Low profitability for smallholders	Producer associations, direct marketing, fair trade models
Regulatory and Safety Hurdles	“Novel food” status, need for toxicological and compositional data for non-traditional parts	Slow approvals, compliance risk	Prepare safety dossiers, harmonize with international regs.
Socio-Cultural Acceptance and Skills	Consumer unfamiliarity outside the Amazon, culinary barriers, limited scale of traditional market	Low consumer uptake	Targeted food innovation, education, tailored product dev.
Infrastructure and Value Chain Gaps	Lack of logistics, post-harvest infrastructure, and continuous supply outside local regions	High costs, product loss	Supply chain investment, regional processing hubs

Source: [4,6,13,15,36,62,63].

## Data Availability

No new data were created or analyzed in this study.

## Abbreviations

The following abbreviations are used in this manuscript:

UAE	Ultrasound-Assisted Extraction
HPP	High-Pressure Processing
HMT	Hydrothermal Treatment
XOS	Xylooligosaccharides
QSAR	Quantitative Structure–Activity Relationship
